# A direct tissue-grafting approach to increasing endogenous brown fat

**DOI:** 10.1038/s41598-018-25866-y

**Published:** 2018-05-21

**Authors:** Nicole R. Blumenfeld, Hwan June Kang, Anna Fenzl, Ziwei Song, Janice J. Chung, Ranjodh Singh, Roshawn Johnson, Ayse Karakecili, Jun B. Feranil, Ninna S. Rossen, Vivian Zhang, Sahir Jaggi, Bret McCarty, Steven Bessler, Gary J. Schwartz, Robert Grant, Judith Korner, Florian W. Kiefer, Brian M. Gillette, Samuel K. Sia

**Affiliations:** 1 Department of Biomedical Engineering, Columbia University, New York, NY 10027 USA; 2 Department of Medicine, Division of Endocrinology and Metabolism, Medical University of Vienna, A-1090 Vienna, Austria; 3 Department of Medicine, Division of Preventative Medicine and Nutrition, Columbia University Medical Center, New York, NY 10032 USA; 4 Departments of Medicine and Neuroscience, Albert Einstein College of Medicine, Bronx, NY 10461 USA; 5 Department of Surgery, Division of Plastic and Reconstructive Surgery, Columbia University Medical Center, New York, NY 10032 USA; 6 Department of Medicine, Division of Endocrinology, Columbia University Medical Center, New York, NY 10032 USA; 7 Department of Chemical Engineering, Ankara University, 06100 Tandogan, Ankara Turkey; 8 Department of Surgery, Division of Wound Healing and Regenerative Medicine, NYU Winthrop Hospital, 259 1st Street, Mineola, NY 11501 USA; 9 Ardent Cell Technologies, Inc., 423 West 127th Street, New York, NY 10027 USA

## Abstract

There is widespread evidence that increasing functional mass of brown adipose tissue (BAT) via browning of white adipose tissue (WAT) could potentially counter obesity and diabetes. However, most current approaches focus on administration of pharmacological compounds which expose patients to highly undesirable side effects. Here, we describe a simple and direct tissue-grafting approach to increase BAT mass through *ex vivo* browning of subcutaneous WAT, followed by re-implantation into the host; this cell-therapy approach could potentially act synergistically with existing pharmacological approaches. With this process, entitled “exBAT”, we identified conditions, in both mouse and human tissue, that convert whole fragments of WAT to BAT via a single step and without unwanted off-target pharmacological effects. We show that *ex vivo*, exBAT exhibited UCP1 immunostaining, lipid droplet formation, and mitochondrial metabolic activity consistent with native BAT. In mice, exBAT exhibited a highly durable phenotype for at least 8 weeks. Overall, these results enable a simple and scalable tissue-grafting strategy, rather than pharmacological approaches, for increasing endogenous BAT and studying its effect on host weight and metabolism.

## Introduction

The obesity epidemic presents significant health and economic risks, afflicting 78 million adults and 13 million children with medical costs estimated to be $150 billion in the U.S. alone^1,2^. Whereas changes in diet and exercise are often ineffective in practice^3^, current weight-loss drugs typically exhibit considerable side effects, are contraindicated in many individuals, and either induce only moderate weight loss or are plainly ineffective in many patients^4,5^. Bariatric surgeries can induce large weight loss and remission of type II diabetes symptoms in the early months, but the procedures are invasive and can produce complications^6^, and weight is often re-gained and up to a third of patients who have remission of type II diabetes symptoms relapse after several years^7^. Thus, there remains an important need to explore new weight-loss approaches with the potential for low rates of complications and long-term efficacy.

Over the past decade, manipulation of brown adipose tissue (BAT) has emerged as a promising approach for treating obesity and metabolic disease^8–28^. BAT is a highly metabolic tissue that exhibits thermogenesis in response to cold exposure via uncoupled respiration mediated by the BAT-specific protein uncoupled protein 1 (UCP1)^29^. BAT consumes large amounts of fatty acids and glucose to support thermogenesis, such that only a small amount of active BAT (~50 grams, a small fraction of typical body weight) can account for 20% of daily energy expenditure^30^. Humans and animals have two major forms of BAT: native BAT, a permanent form present at distinct anatomic sites (i.e. between anterior neck muscles, under the clavicle, surrounding the aorta and other vasculature, etc^31^) and inducible BAT (also called beige or brite fat), which can develop within WAT in response to endogenous or exogenous stimuli, in a process referred to as browning^8–14,32–37^.

There is widespread evidence in animals that increasing the mass or activity of BAT or beige fat has enormous potential to prevent and/or reverse obesity and metabolic disease^14^. While several approaches have been proposed for increasing BAT mass or activity in humans, significant drawbacks have prevented them from reaching the clinic. First, cold exposure has resulted in reductions in body fat mass and changes in glucose and insulin sensitivity^38–42^, but long-term cold exposure is nearly impossible to implement for most patients. Second, several classes of drugs (such as sympathomimetic drugs and β3-adrenergic agonists) have been shown to increase BAT activity in animals and humans; however, these drugs have been found to exert unwanted off-target effects, including potentially life-threatening cardiovascular effects, limiting their safety and efficacy^16,39,43^. Third, while differentiation of patient-derived adipocyte progenitors for transplantation is a potentially promising strategy to increase BAT mass, this method poses significant manufacturing challenges due to the procedural complexity involved in isolation, expansion, and differentiation of autologous adipose precursor cells for individual patients^23,24,44–46^. The lack of vascularization and other tissue components may also limit the viability and function of large amounts of transplanted cells^47^.

In this study, we explore a novel and direct approach for increasing long-term BAT mass *in vivo*, by isolating host WAT, conversion of whole adipose tissue fragments to BAT-like fragments in a single *ex vivo* step, followed by implantation of the converted BAT. This method is simple to perform, can be easily scaled, eliminates the danger of off-target pharmacologic effects, and retains the native vascularization and tissue microenvironment important in maintaining BAT functionality, and thus enables metabolic characterization of hosts with tissue-grafted converted BAT.

## Results

### Concept of exBAT

**Figure 1:**
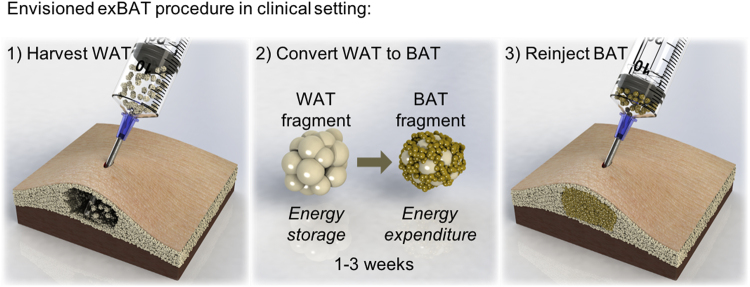
Concept of tissue-engineering therapeutic approach to increase endogeneous brown fat via a single-step *ex vivo* browning method. Illustration of 3-step process for increasing brown adipose tissue (BAT) in humans through *ex vivo* browning: (1) subcutaneous white adipose tissue (WAT) is harvested by liposuction or excision and cultured as tissue fragments; (2) WAT fragments are exposed to chronic browning stimuli (i.e. browning factors in the media) to convert the WAT to BAT, in a process that takes approximately 1 to 3 weeks; (3) the converted BAT fragments are autologously reimplanted within WAT.

The three steps of exBAT are: 1) harvesting of host WAT; 2) exposure of WAT fragments to browning factors via single-step culture; and 3) re-implantation of converted BAT within subcutaneous WAT (Fig. 1). While only small amounts of BAT can have a significant impact on metabolism^48^, large amounts of viable WAT can be obtained by plastic surgeons using well-established harvesting techniques (i.e. liposuction)^49,50^. Moreover, compared to traditional processes that involve sorting and purification of isolated progenitor cells followed by long periods of cell-culture expansion, the exBAT procedure is quick, because a single browning step acts on whole tissue fragments to convert WAT to BAT mass, which is ready for direct implantation.

### Development and characterization of *ex vivo* browning process in mice

**Figure 2:**
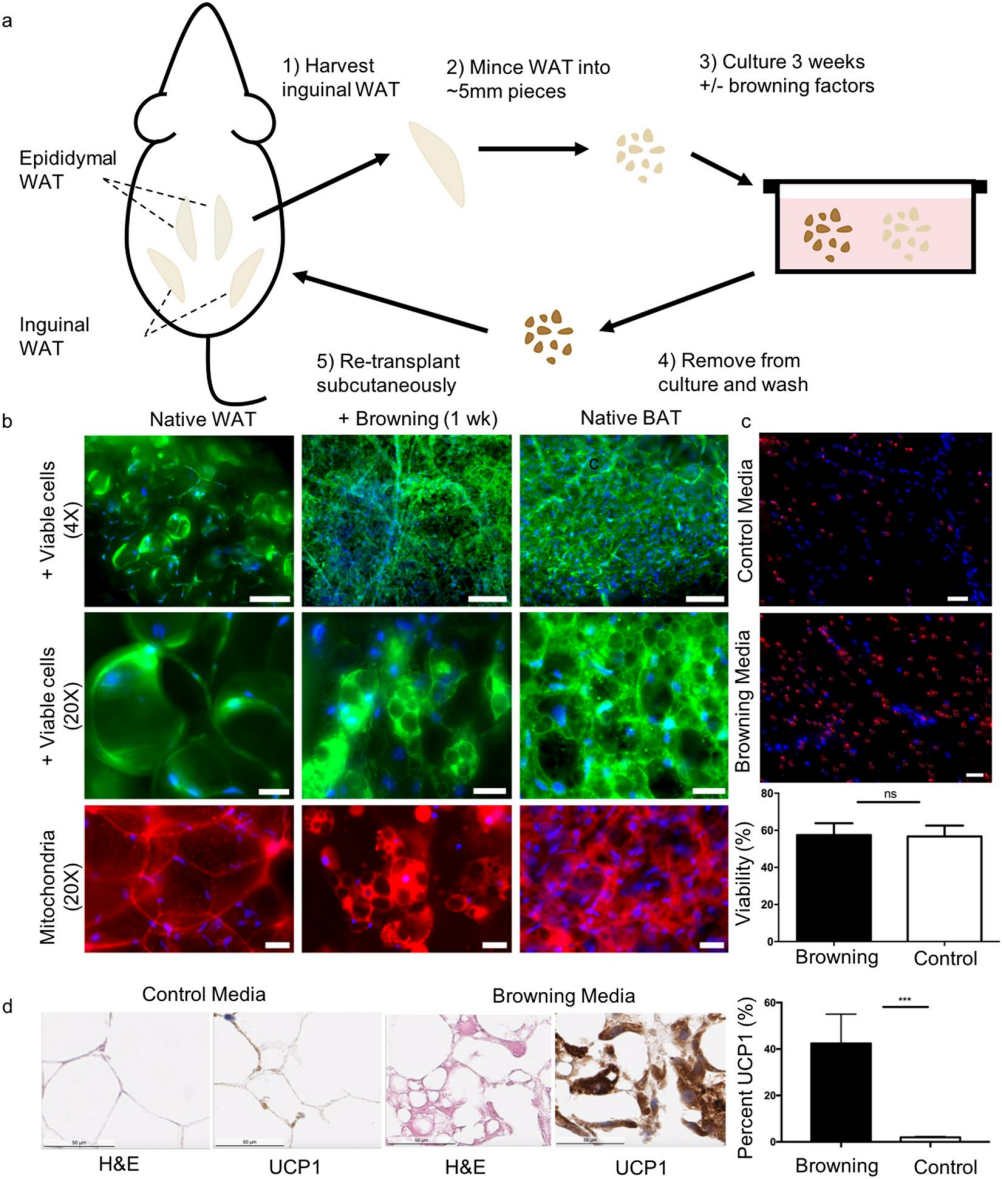
Single-step browning of mouse WAT tissue *ex vivo*. **(a)** Illustration of experimental design for studies of *ex vivo* browning and autologous transplantation in mice: (1) subcutaneous WAT from the left inguinal depot is excised from anesthetized mouse; (2) WAT fragments are gently minced into 2–5-mm fragments; (3) WAT fragments are cultured in media for 1–3 weeks in control or browning media; 4) fragments are removed from media and washed with PBS; (5) fragments are re-implanted subcutaneously adjacent to the right inguinal WAT depot. **(b)** Live-cell and mitochondrial staining of inguinal WAT fragments both immediately after harvest (left) and one week of culture with browning factors (middle); native interscapular BAT fragments immediately after harvest (right). Epifluorescence images show staining for calcein AM (green, indicates cytoplasm in live cells), Mitotracker (red, indicates active mitochondria in live cells), and Hoescht (blue, indicates nuclei). Scale bars are 150 µm (top row) and 30 µm (middle and bottom rows). **(c)** Dual live-dead staining after three weeks in culture. Top panel shows representative image of tissues cultured in control media and middle panel shows a representative image of tissues cultured in browning media (scale bar 100 is µm for both). Ethidium homodimer (red) labels dead nuclei and Hoescht (blue) labels all nuclei. Bottom panel shows quantification of live/dead ratio for tissues cultured in browning media and control media for 3 weeks. Graphs display Mean +/− SEM; n = 7 for browning media, n = 11 for control media (each sample came from independent culture experiment); *p* = 0.9364 using Student’s t-test. **(d)** H&E staining and UCP1 immunohistochemistry of inguinal WAT fragments cultured in control or browning media for 10 days. Scale bars are 50 µm. Quantification of percent UCP1 per tissue area, quantified by automated analysis of DAB positive areas in images of 10-micron sections (*p* < 0.001, Student’s t-test), is shown on the right. Error bars show SEM (n = 10).

We first developed a mouse model to test whether whole WAT fragments could be converted to BAT *ex vivo* (Fig. 2a). We first excised a small piece (~0.5 mL) of subcutaneous WAT from the left inguinal depot, located along the rear flank of the mouse above the hindlimb. Next, we gently minced the WAT tissue into fragments of approximately 2 to 5 mm in diameter to mimic the size of fragments obtained during fat harvesting procedures in humans and suspended the fragments in either browning media or control media (i.e. basal media without browning factors). Working with whole pieces of tissue is simpler than working with individual cell populations such as adipocyte progenitors, which are frequently isolated from adipose tissue depots and require expansion in 2D culture^9,16^. We used a cocktail of rosiglitazone (PPARγ agonist), isobutylmethylxanthene (IBMX, phosphodiesterase inhibitor), T3 (thyroid hormone), indomethacin (COX inhibitor), CL316,243 (β3 adrenoreceptor agonist), and vascular endothelial growth factor (VEGF) (see Methods for details). This single-step cocktail was found to induce browning over a duration of 1–3 weeks *ex-vivo* while maintaining cell viability, compared to multi-step induction which was previously performed for browning of adipocyte progenitors^51,52^.

We performed live-cell staining on whole tissue fragments cultured in the presence of browning media (Fig. 2b,c). Initially, the WAT fragments displayed cytoplasmic and mitochondrial staining around large lipid droplets, as well as in branching vascular structures (Fig. 2b, left panel). After one week in browning media, tissues displayed higher cell density with smaller and increased number of lipid droplets, consistent with formation of BAT-like tissue (Fig. 2b, middle and right panels). Assessment of cell viability, which could affect the efficacy of the implant *in vivo*, showed that viability after 3 weeks of *ex vivo* browning was comparable to that of control media under the same duration (Fig. 2c).

To further test the extent to which we could achieve *ex vivo* browning from subcutaneous WAT fragments, we performed immunohistochemistry on samples that had been cultured in both browning and control media for 10 days. Samples cultured in BAT media exhibited smaller lipid droplets and significantly higher UCP1 staining (Fig. 2d, *p* < 0.001), consistent with what would be expected of native BAT.

### Autologous re-implantation into mice and persistence of BAT-like phenotype *in vivo*

Next, we examined whether the BAT-like properties of tissues cultured in browning media would persist weeks after re-implantation. After 3 weeks of *ex vivo* culture, we subcutaneously implanted approximately 0.2 mL of autologous converted BAT on the right inguinal WAT depot and extracted the tissue again from the implant site after 8 weeks.

**Figure 3:**
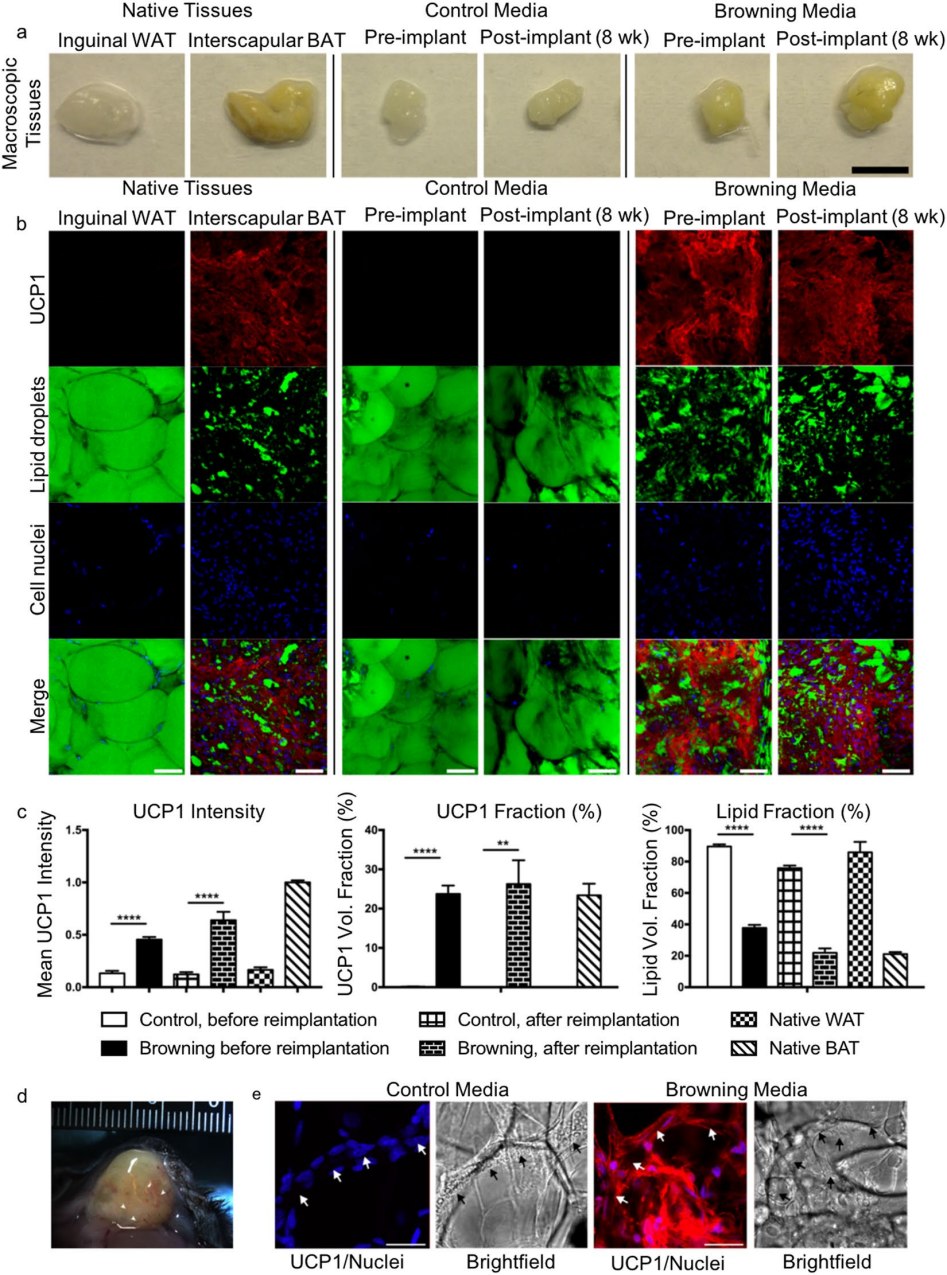
Autologous re-implantation of converted BAT, and analysis after 8 weeks in mice. Experimental design is shown in Fig. 2a. (**a**) Macroscopic images of native inguinal WAT, native interscapular BAT, and WAT fragments that were cultured for three weeks in browning or control media, imaged before (pre) and after (post) 8 weeks re-implantation. Scale bar is 3 mm. **(b)** Confocal microscopy of native inguinal WAT, native interscapular BAT, and WAT fragments that were cultured for three weeks in browning or control media, imaged before (pre) and after (post) re-implantation. Scale bars are 50 µm. Images stained for UCP1 expression (red), and counterstained with LipidTox (green) and Sytox nuclear stain (blue). **(c)** Mean UCP1 intensity (left), UCP1 volume fraction (middle), and lipid volume fraction (right) measurements from 3D confocal images of whole-mount stained tissues after 3 weeks of culture (pre-implant) and 8 weeks after reimplantation (post-implant). Error bars indicate SEM. Compared to control media, UCP1 intensity for fragments cultured in browning media was significantly higher both before (*p* < 0.0001) and after (*p* < 0.0001) reimplantation, as determined by one-way ANOVA and Bonferroni post hoc tests. UCP1 volume fraction (middle) and lipid volume fraction (right) were also statistically significant both before (*p* < 0.0001 and *p* < 0.0001) and after (*p* = 0.0012 and *p* < 0.0001) reimplantation. **(d)** Image of reimplanted tissue that was cultured for 3 weeks in browning media, 8 weeks following reimplantation. The implanted tissue formed a fat pad that became vascularized (arrowheads) within the surrounding subcutaneous WAT. **(e)** High magnification confocal images of tissues cultured in both control media (left) and browning media (right), showing channel networks of putative capillaries within explanted tissues. Scale bars are 30 µm. Images stained for UCP1 expression (red) and counterstained with Sytox nuclear stain (blue); greyscale displays transmitted light.

After 8 weeks of implantation, tissue fragments cultured in control media during the *ex vivo* process retained a WAT-like appearance (Fig. 3a). However, we observed that tissues cultured in browning media during the *ex vivo* period, after 8 weeks of implantation, continued to exhibit a brown color consistent with BAT which contains a high density of iron-rich mitochondria (Fig. 3a). We performed immunostaining for UCP1 (with counterstaining to label lipid droplets and cell nuclei) on both whole-tissue fragments immediately before re-implantation and on extracted tissue after 8 weeks of implantation. As negative and positive controls, native uncultured inguinal WAT and interscapular BAT tissue fragments were also stained (Fig. 3b, left panels). Tissues cultured in control media prior to re-implantation retained a WAT-like appearance (Fig. 3b, middle panels). By comparison, tissues cultured in browning media exhibited high levels of UCP1 signal, numerous small lipid droplets, and a high density of cell nuclei (Fig. 3b, right panels), both in pre-implant whole-tissue fragments and extracted tissues after 8 weeks of implantation.

Using 3D confocal imaging and segmentation of image stacks through whole-mount stained tissues (Supplementary Fig. 1 and Videos 1–4), we quantified UCP1 immunostaining intensity, UCP1 volume fraction, and lipid volume fraction before and after re-implantation (Fig. 3c). Compared to tissues in the control media, tissues cultured in the browning media exhibited significantly higher UCP1 intensity, significantly higher UCP1 volume fraction, and significantly lower lipid volume fraction, both before and after implantation (Fig. 3c). These trends were consistent with phenotypes of the converted BAT in both pre- and post-implant states, and consistent with what we observed for native BAT vs. WAT phenotypes.

Finally, within the whole-tissue fragment, we also observed functional blood vessels as indicated by red blood cell-filled vessels that were visible by eye in the grafted tissue (Fig. 3d). The intact vascular structures within the engrafted converted exBAT were also visible under epifluorescence and brightfield microscopy after 8 weeks of re-implantation (Fig. 3e).

### Successful conversion of human subcutaneous WAT into exBAT

**Figure 4:**
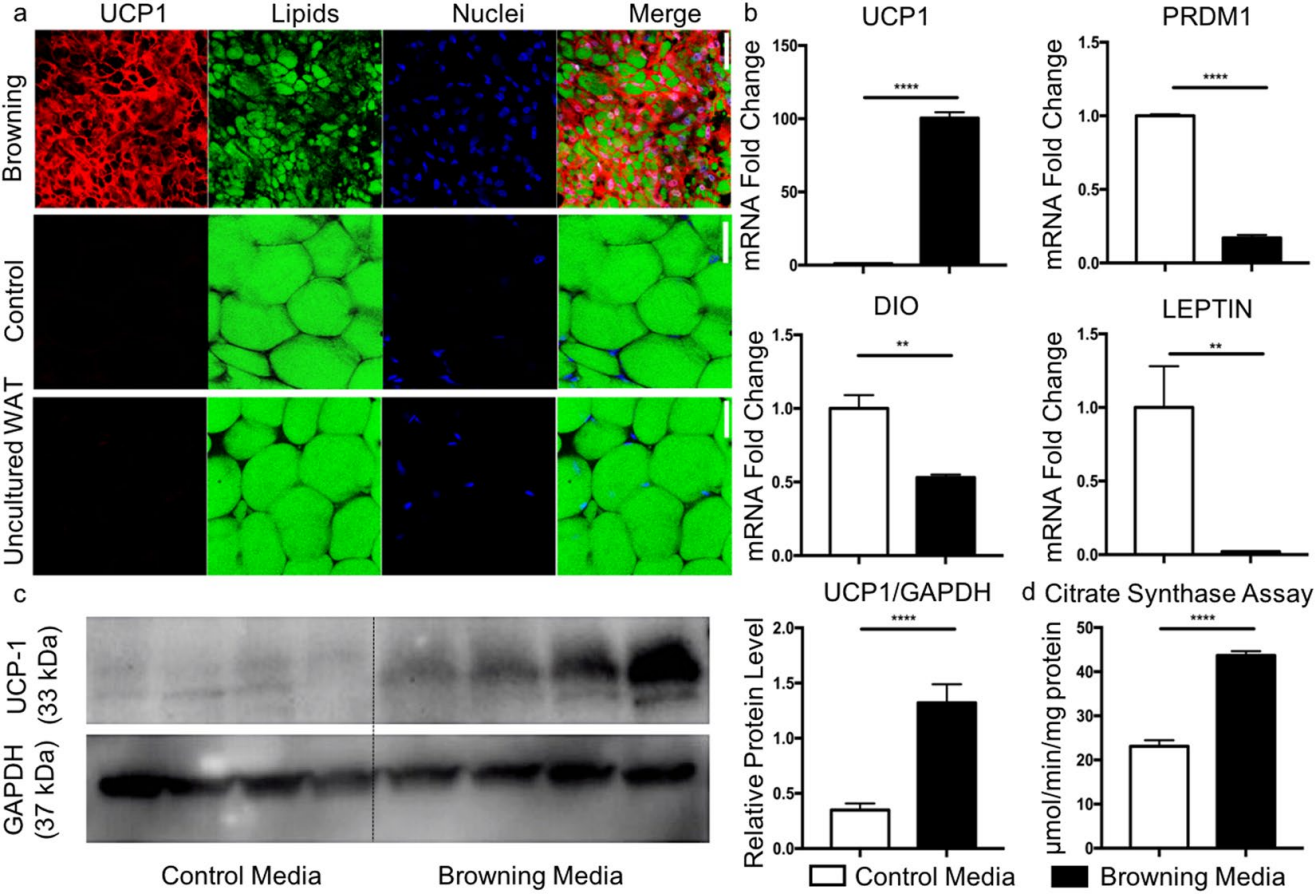
Single-step browning of human WAT tissue *ex vivo*. Experimental design was shown in Fig. 1. (**a**) Human WAT fragments cultured in browning media (top panel) and control media (middle panel) for 3 weeks and native human WAT (not cultured, bottom panel). Tissue was stained for UCP1 expression (red) and counterstained with LipidTox (green) and Sytox nuclear stain (blue). Scalebars are 50 µm. (**b)** RNA expression levels in human fragments cultured in control versus browning media. Left to right: UCP1, PRDM16, DIO and LEPTIN. **(c)** Western blot analysis of UCP1 protein expression in human WAT fragments cultured in control (left) or browning (right) media for 2 weeks. Protein expression is normalized to GAPDH. Compared to control tissues, UCP1 protein expression in tissues cultured in browning media was significantly greater (*p* < 0.0001). **(d)** Citrate synthase analysis of UCP1 activity in control media and browning media. Student’s t-test performed (*p* < 0.0001).

We next sought to investigate whether human subcutaneous WAT could also be converted to BAT with our single-step *ex vivo* browning method. For these studies, we collected excess subcutaneous WAT samples via fat harvesting from the abdominal region of patients who underwent autologous fat grafting procedures (see Materials and Methods for details). Tissues were cultured in the same media and with the same browning factors as previously described for mouse tissues. After 3 weeks of *ex vivo* culture, we observed that human WAT tissue fragments cultured in browning media developed significant UCP1 expression and smaller lipid droplets (Fig. 4a, top panel, Supplementary Fig. 2d–f). Tissues cultured in control media retained a WAT-like phenotype, similar to native human WAT that was not cultured (Fig. 4a, middle and bottom panels). Immunohistochemistry demonstrated significantly increased UCP1 intensity, UCP1 volume fraction, and significantly decreased lipid volume fraction in exBAT cultured in browning compared to control conditions, comparable with the expression in native tissues (Supplementary Fig. 3). We assessed mRNA expression via qPCR for a variety of genes known to be differentially expressed in native BAT relative to WAT. In the converted exBAT, UCP1 mRNA levels were preferentially upregulated, while DIO2, leptin, and PRDM16 mRNA levels were downregulated (Fig. 4b), concordant with previous reports of human tissues *in vitro*^53^. Accordingly, UCP1 protein content (Fig. 4c), and citrate synthase activity (Fig. 4d), a measure of mitochondrial metabolic activity, were significantly increased under browning conditions, indicating that human exBAT possess not only cellular and molecular but also functional characteristics of thermogenically active fat (*p* < 0.0001).

### Allogeneic transplantation of exBAT in mice with diet-induced obesity

**Figure 5:**
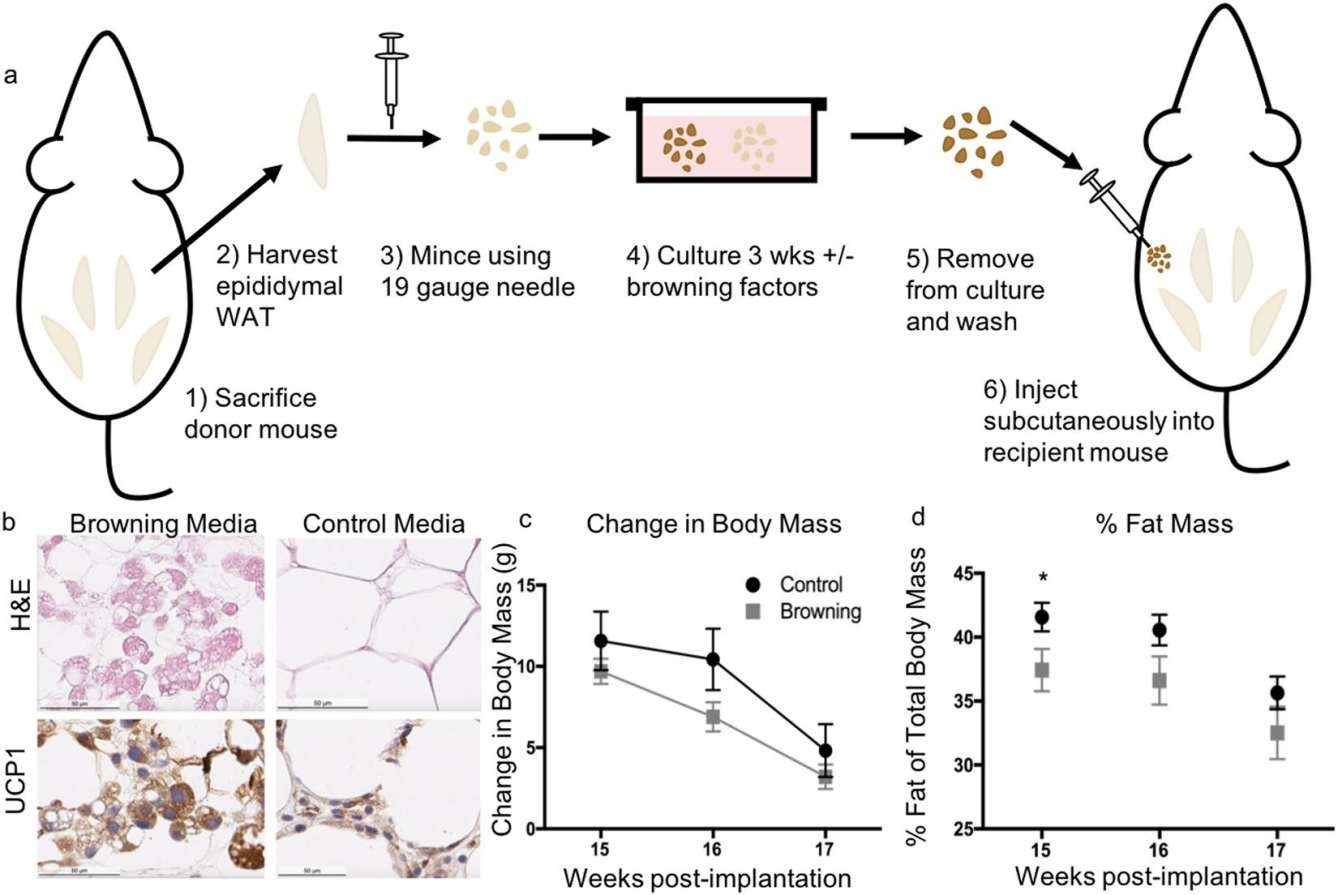
Metabolic testing of reimplanted exBAT in mice. **(a)** Illustration of experimental design for studies of *ex vivo* browning of epididymal WAT and allogeneic injection (the allogeneic implantation was designed to minimize stress and background weight loss of mice): (1) donor mouse is sacrificed via CO_2_ asphyxiation; (2) epididymal WAT fragments are harvested from sacrificed donor mouse via necropsy; (3) WAT fragments are minced by passaging through a 19 gauge needle; (4) WAT fragments are cultured for 3 weeks in media with and without browning factors; (5) fragments are removed from media and washed with PBS; (6) fragments are injected subcutaneously into age-matched, sex-matched recipient mouse. **(b)** H&E (top) and UCP1 IHC (bottom) staining of cultured tissues (10 days) prior to implantation (note: visceral adipose tissue). Scale bar: 50 µm. **(c)** Change in body mass 15–17 weeks post-implantation (see Supplementary Fig. 4 for entire weight-loss data). Drop in body weight is due to entry into metabolic chambers from week 15–16, and subsequently exposure to cold temperature from week 16–17. Error bars indicate SEM, n = 8 for each group. **(d)** Percent fat mass of total body mass for control and experimental groups. Percent fat mass is measured prior to entering the metabolic chambers (15 weeks post-implant, *p* = 0.056), after 1 week in the chambers at 25 °C (16 weeks post-implant), and after 1 more week in the chambers at 8 °C (17 weeks post-implant). Error bars indicate SEM, n = 8 for each group.

Finally, we performed preliminary metabolic phenotyping of exBAT transplantation in a diet-induced obesity (DIO) mouse model to mimic the potential target population in humans. Here, we used allogeneic transplantation, performed in previous DIO mouse models^54–58^, to minimize variations in stress and weight caused by the multiple surgeries of autologous re-implantation (Fig. 5a). To mimic the envisioned clinical procedure in humans, we developed a minimally invasive delivery method, injecting the converted browned tissue via a 19-gauge (1.1 mm × 25 mm) needle into the subcutaneous, lower dorsal region of recipient mice. For donor tissue, we used visceral WAT from epididymal regions of age- and sex-matched DIO mice which is elevated in obese human subjects^25^ and exhibits soft mechanical properties (compared to subcutaneous WAT) which made it suitable for needle injection; although epididymal WAT had previously not been as well demonstrated to convert to BAT as inguinal WAT, we found it to brown effectively using our single-step *ex vivo* method (Fig. 5b, Supplementary Fig. 4). As a control, we also injected visceral WAT cultured in control media, denoted “control WAT”. We injected ~0.2 mL of cultured tissue fragments, which was the yield from one donor fat pad; in this study, we tried just a single dosage to perform a preliminary assessment of the *in vivo* effect before a full-scale trial of therapeutic dosages and conditions in a future trial.

Throughout 17 weeks post-implantation, mice injected with control WAT and the converted BAT both exhibited weight losses after being moved to the metabolic chamber and after cold exposure, as expected. Mice receiving converted BAT exhibited lower body weight than those injected with control WAT throughout the entire 17 weeks in terms of mean weights, but no statistical significance was observed (Fig. 5c, Supplementary Fig. 5a). EchoMRI measurements of percent fat mass of the animals (Fig. 5d) were taken prior to entry into metabolic chambers, after 1 week in the chambers at room temperature, and after 1 more week in the chambers at 8 °C, and approached significant difference at 15 weeks post-implant (Fig. 5d, *p* = 0.056). Differences in VO_2_ and heat expenditure in mice 1 week following implantation were non-significant (Supplementary Fig. 5b,c). Changes in dosage or cell source could be tested in future experiments.

## Discussion

We have demonstrated a novel tissue-engineering approach for directly increasing functional BAT mass in a host in large amounts. Our method of increasing endogenous brown fat contrasts with traditional approaches based on systemic pharmacological agents, stem-cell differentiation, or prolonged cold exposure, which have shown promise in increasing BAT mass but have significant drawbacks, such as off-target effects and impractical clinical use. Unlike isolated cells in 2D culture, whole-tissue fragments retain multiple cell types, 3D extracellular matrix scaffolding, and intact cellular niches, giving rise to an additional potential advantage of maintaining physiological phenotype *in vivo*. Consistent with previous studies suggesting several cell types and tissue structures to be involved in native browning^59^, we observed that, beyond adipocytes, vessel-like structures appeared to remain viable during *ex vivo* culture and that tissue fragments became re-vascularized after re-implantation. While further studies are needed to delineate the molecular pathways governing the BAT transplants, the ability to culture large quantities of tissue at once while retaining 3D structure, including vascular cells and structures, holds translational clinical utility, and has typically not been possible through the culture of isolated adipocyte progenitor cells^60,61^.

One barrier to the use of engineered BAT as a therapeutic for countering obesity and metabolic disorders is the scalability of traditional cell- and tissue-engineering approaches. The exBAT method is simple and practical to perform. The browning factors used in our media comprise approved drugs or endogenous factors (it should be noted that FBS was used in our browning media, however, this could be replaced with human serum in a clinical setting), enhancing safety in case of trace amounts following re-implantation^16^. Moreover, the method requires only a single-step culture period of whole WAT tissue fragments (without the need to isolate and purify individual cell populations) to produce BAT-like tissue. The approach eliminates the need for technically challenging stem-cell isolation and expansion, which can be complex to implement at scale. This single-culture step could be easily implemented into existing autologous fat grafting procedures, and potentially even production within the clinician’s office (as has been achieved for adipose stem cell-based therapies^62^).

While metabolic phenotyping data was only conducted with a single dosage in this study, future variations of procedural modifications with increased dosage variations could be tested. Additional studies could be performed to further assess changes in cell viability over time. Finally, the use of autologous instead of allogeneic implantations, and the use of inguinal adipose tissue may help produce significant *in vivo* results.

For future studies, we note that the amount of native BAT present in humans, correlated with leanness and metabolic health, is in the range of 50 to 100 mL, and an average of 70 mL of native BAT has been shown to significantly increase whole-body energy expenditure, glucose and fatty acid metabolism^41^. Since large amounts of excess WAT can be harvested from overweight and obese patients via traditional fat-grafting procedures (i.e. liposuction), and small amounts of BAT can have a large impact on metabolism, direct *ex vivo* browning of WAT could generate sufficient BAT mass to test metabolic benefits more thoroughly than this study. Overall, a tissue-grafting approach to increase endogenous levels of brown fat could present a complementary method, alongside current approaches to reduce energy intake (such as appetite suppressing drugs and surgeries) or pharmacology-based approaches to increasing brown fat, in order to address the significant clinical need for effective therapies for obesity and diabetes.

## Methods

### Preparation of browning and control media

The control media was prepared by adding 10% fetal bovine serum (FBS), 1% Penicillin Streptomycin, 20 mM HEPES, 50 μg mL^−1^ sodium ascorbate, 1 μM insulin into Dulbecco’s Modified Eagle Medium (DMEM, high glucose, GlutaMAX Supplement, pyruvate; 10569010, ThermoFisher). The browning media was prepared by adding 1 μM Dexamethasone, 500 μM Isobutylmethylxanthine, 50 μM Indomethacin, 1 μM Rosiglitazone and 1 μM CL316243, and 250 nM triiodothyronine (T3), and 25 ng mL^−1^ VEGF into control media.

### Mouse WAT harvesting, processing, and culture (native inguinal WAT) for autologous re-implant-ation experiments (Figs 2 and 3)

All animal procedures were approved by the Columbia University Institutional Animal Care and Use Committee (IACUC) and all experiments were performed in accordance with relevant guidelines/regulations. The mice used in this study were the C57BL/6 strain (Taconic) and were maintained on a high fat diet (Research Diets) starting at 6 weeks of age. Mice were 22 weeks of age at the time of the initial surgery to extract WAT. During surgical procedures, mice were anesthetized with isoflurane and maintained on a warm water circulating pad to maintain temperature. To extract inguinal WAT, a small 1 cm incision was made on the midline of posterior dorsum adjacent to the inguinal depot. The inguinal WAT depots extend from the dorsolumbar region to the gluteal region of the mouse and are anatomically distinct from surrounding tissue and muscle^63^. Skin was gently lifted and retracted and a portion of the inguinal WAT depot (approximately 0.3–0.5 grams) was lifted with forceps and excised using a scalpel. Tissues were transferred aseptically to tubes containing phosphate buffered saline (PBS) and weighed. The incisions were closed with wound clips and mice were allowed to recover with a pain-relieving drug (buprenorphine, 5 mg/kg) administered for two days.

After briefly rinsing the explanted tissues in PBS, the tissues were transferred into DMEM, gently cut into small pieces (~2–5 mm) using #11 scalpel blades and rinsed 3 times in 30 mL PBS at 37 °C. Tissue culture flasks (75 cm^2^) were used to incubate the tissues and 12 mL of each type of media was added into each flask. The adipose tissues were incubated in a 37 °C incubator with 5% CO_2_ and the culture media were changed every two days for a duration of three weeks. After the culture period, a portion of the live tissue fragments were used for viability and mitochondrial staining and another portion of the tissues were fixed in 4% paraformaldehyde (PFA) for 24 hours and stored in PBS for immunostaining. The rest of cultured tissues (approximately 0.2–0.3 grams) were reimplanted into the same mice where they were initially explanted. 8 weeks after reimplantation, the animals were euthanized by carbon dioxide inhalation, and the tissues were taken out and fixed in 4% PFA for 24 hours and stored in PBS. Implanted tissues were able to be identified by the naked eye (Fig. 3d) as they do not fully integrate, and no translocation was observed.

Native interscapular BAT and inguinal WAT were also excised from euthanized mice to serve as control tissues in staining procedures. The control tissues were not cultured in any media and they were fixed in 4% PFA immediately after explantation for 24 hours and stored in PBS.

### Viability and mitochondrial staining

To perform viability and mitochondrial staining, we transferred several tissue fragments per mouse into 96 well plates, and then incubated for 1 hour at 37 °C in a solution of DMEM with Calcein AM (Life Technologies) to label cytoplasm in live cells, NucBlue Live Cell Stain ReadyProbes reagent (Life technologies, Hoechst 33342 Special Formulation) to label nuclei, and MitoTracker Deep Red FM (Life Technologies) to label active mitochondria. Fragments were then rinsed in DPBS, transferred to a glass slide, sandwiched under a coverslip, and then imaged immediately using a Leica DMi6000b widefield fluorescent microscope. For dual live/dead staining, dead nuclei were stained using Ethidium homodimer and all nuclei were stained using Hoescht blue. Cells were imaged using a Leica DMi6000b widefield fluorescent microscope. Manual counting was performed on maximum intensity projection images of z-stacks (10X magnification), using the Leica LAS AF software.

### H&E and IHC staining and quantitative analysis

Following dissection or after a specified culture period, tissues were rinsed 3X in PBS and subsequently fixed in 10% neutral buffered formalin overnight at room temperature. Tissues were paraffin embedded, sectioned, and stained by HistoWiz Inc (Brooklyn, NY). To quantify percent UCP1/tissue area and mean lipid droplet area, we used Aperio Imagescope (Leica Biosystems) to acquire representative 10x images from each IHC stain for WAT and BAT inguinal tissue, and processed those images through automated segmentation and measurement of DAB positive areas using an ImageJ macro (available upon request).

### Whole mount staining of adipose tissue fragments

Following dissection or after a specified culture period, tissue fragments were fixed in fresh 4% paraformaldehyde solution in phosphate buffered saline (PBS) overnight, then transferred to PBS. The fixed whole mount adipose tissues were stained with anti-UCP1 antibody produced in rabbit (U6382, Sigma-Aldrich), based on the protocol described by Xue *et al*.^64^, which was optimized for the best result in our study (see Supp. Experimental Procedures for details).

### Fluorescent imaging and quantitative analysis

Fluorescent images were acquired using Leica TCS SP5 confocal microscopes using LAS AF software (Leica Microsystems). We maintained consistent exposure settings across different samples and acquired wide-area z-stacks across tissue fragments using an automated stage (Supplementary Videos 1–4). To quantify UCP1 intensity for individual samples, we calculated the mean UCP1 intensity across all z-slices. Multiple tissue fragments were quantified for each condition, and controls without primary antibody were used to normalize for background fluorescence. To quantify UCP1 and lipid volume fraction for individual samples, we binarized each image in the z-stack with Otsu’s method while taking into account the slightly dimmer signal in the deeper slices using a custom-written MATLAB script (available upon request). We visually checked this binarization for each image in all the z-stacks (Supplementary Fig. 1). Volume fractions were then calculated from the binarized z-stacks, the top-half and bottom-half volumes being comparable within each z-stack.

### Allogeneic injection of adipose tissue fragments

All animal procedures were approved by the Columbia University Institutional Animal Care and Use Committee (IACUC) and all experiments were performed in accordance with relevant guidelines/regulations. The mice used in this study were the C57BL/6 strain (Taconic) and were maintained on a high fat diet (Research Diets) starting at 6 weeks of age. Donor mice (12 weeks old) were sacrificed using CO_2_ asphyxiation, and visceral epididymal fat pads were dissected. Both fat pads were harvested from each donor mouse; one for culture in control media and one for culture in browning media. Fat pads were placed in DMEM and rinsed 3x with PBS. Each fat pad was gently broken up into 2–5 mm pieces using forceps to mimic the size of fragments obtained by traditional fat harvesting techniques, which cannot be easily performed on small reservoirs of fat in mice^58^. The pieces were then passed through a sterile 19-gauge needle to further break up the pieces. Fat was cultured for three weeks using the same media as described previously with media changes once every 2 days. After 3 weeks, fat was rinsed 3x in PBS and ~0.2 mL minced fat was loaded into a sterile 19-gauge needle and subcutaneously injected above the right rear hindlimb. Mice were allowed to recover for 1 week, and were monitored for any adverse reactions.

### Metabolic phenotyping (indirect calorimetry and MRI)

Following the injection, mice were transferred to the mouse metabolic phenotyping core facility at Columbia University and allowed to acclimate for 1 week. Mice were kept un a high-fat diet (Research Diets) beginning at 6 weeks of age and maintained on this diet throughout the duration of the experiment. Food and cages were changed weekly and body weights were measured weekly.

Comprehensive Lab Animal Monitoring System (Oxymax/CLAMS, Columbus Instruments) was used for metabolic phenotyping at 1, 11, and 15 weeks post-implantation (see Supp. Experimental Procedures for details).

We used EchoMRI-100H (EchoMRI™; Echo Medical Systems) to measure percent fat mass of the mice (see Supp. Experimental Procedures for details). Measurements were taken before placement in the metabolic chambers, after one week in the chambers at room temperature, and after one week in the chambers at 8 °C. This sequence of MRI measurements was repeated each of the three runs in the metabolic chambers (i.e. 1, 11, and 15 weeks post-implantation).

### Human specimens

Human subcutaneous adipose tissue samples were obtained under a protocol approved by the Columbia University Medical Center IRB for research use of de-identified discarded tissue from autologous fat transfer procedures (Columbia University Medical Center Protocol #AAAN7112) and all experiments were performed in accordance with relevant guidelines/regulations. Patients reviewed and signed informed-consent documents describing the use of their discarded adipose tissue and de-identified data for research into browning of adipose tissue. Samples were obtained from the abdominal region using standard fat harvesting procedures, which involved local injection of tumescent anesthesia for 15 min followed by manual harvesting using a 3 mm cannula and 20 cc syringe. Approximately 6 cc of leftover tissues were retrieved within 4 hours of removal, transferred to a biosafety cabinet, rinsed 3 times with 100 mL DPBS, then transferred to culture. The adipose fragments were approximately 1–3 mm in size and did not require further mincing or processing for culture.

### Human citrate synthase assay, Western blot, qPCR

Citrate synthase activity assays to assess tissue mitochondrial function were performed as described previously^33^. Protein lysate (8 μg) was used for the citrate synthase activity assay following the manufacturer’s protocol (Sigma CS070). Tissue samples were homogenized in tissue lysis buffer provided with the kits to obtain protein lysates. After centrifugation and removal of the lipid layer, protein concentration was determined using the BCA Protein Assay (Thermo Fisher Scientific Inc.).

For gene expression assays, total RNA was extracted using Trizol (Invitrogen), treated with DNase (ThermoScientific) and reverse transcribed to cDNA (AppliedBiosystems) according to manufacturer’s instructions. Gene expression, normalized to 36B4, was analyzed by quantitative real-time RT-PCR (Sybr Green, 384-well plates) using the QuantStudio 6 PCR System (ThermoFisher). Primer sequences are available upon request.

To perform immunoblot analysis, homogenized tissue was lysed in protein lysis buffer (Sigma) containing protease and phosphatase inhibitors. Standard western blotting was performed using rabbit polyclonal antibodies to Ucp1 (1:500; Abcam; ab23841) and GAPDH (1:3000; CellSignaling; #2118). HRP linked Goat anti-Rabbit (1:3000; Biorad; #170–6515) was used as secondary antibody. Proteins were detected by chemiluminescence (Roche) and images were acquired using FusionFx (Peqlab).

### Statistical analysis

One and two-way ANOVA tests and Bonferroni/Tukey post hoc tests were performed using Graphpad Prism 7 software. Student’s t-test was performed using Graphpad Prism 7 software.

## Electronic supplementary material

Supplementary Information

Video S1

Video S2

Video S3

Video S4
