# Supplementary material for: A direct tissue-grafting approach to increasing endogenous brown fat
Source: Sci Rep. 2018 May 21;8:7957. doi: 10.1038/s41598-018-25866-y (PMC5962549; doi:10.1038/s41598-018-25866-y)

A direct tissue-grafting approach to increasing endogenous brown fat

Nicole R. Blumenfeld[a], Hwan June Kang[a], Anna Fenzl[b], Ziwei Song[a], Janice J. Chung[a], Ranjodh Singh[a], Roshawn Johnson[a], Ayse Karakecili[a,1], Jun B. Feranil[c], Ninna S. Rossen[a], Vivian Zhang[a], Sahir Jaggi[a], Bret McCarty[a], Steven Bessler[a], Gary J. Schwartz[d], Robert Grant[e], Judith Korner[f], Florian W. Kiefer[b], Brian M. Gillette[a,2,3,*] and Samuel K. Sia[a,*]

[a]Department of Biomedical Engineering, Columbia University, New York, NY 10027, USA.

[b]Department of Medicine, Division of Endocrinology and Metabolism, Medical University of Vienna, A-1090 Vienna, Austria.

[c]Department of Medicine, Division of Preventative Medicine and Nutrition, Columbia University Medical Center, New York, NY, 10032, USA.

[d]Departments of Medicine and Neuroscience, Albert Einstein College of Medicine, Bronx, NY, 10461, USA.

[e]Department of Surgery, Division of Plastic and Reconstructive Surgery, Columbia University Medical Center, New York, NY 10032, USA.

[f]Department of Medicine, Division of Endocrinology, Columbia University Medical Center, New York, NY, 10032 USA.

SUPPLEMENTARY INFORMATION:

Supplementary Methods:

Whole-mount staining of adipose tissue fragments: On day 1, the whole adipose tissues stored in PBS were cut into smaller pieces (2mm x 2mm) and transferred into a 24-well plate. The tissue fragments were briefly washed with PBS, treated with proteinase K (5 min) to break down extracellular matrix and incubated with 100% methanol for permeabilization of the tissues (30min). 3% Blocking buffer solution was prepared by adding non-fat dried milk power (1.5g) into 1% Triton X-100 (Sigma Aldrich) solution (50ml) in PBS and tissues were incubated with the blocking buffer overnight (12 – 24hr) at 4°C on a rocking board. On day 2, the tissues were washed with 1% Triton X-100 solution for 15 minutes and incubated with the primary UCP1 antibody diluted 1:200 in 3% blocking buffer overnight at 4°C (12 – 24hr) on a rocking board. On day 3, the tissues were washed with 1% Triton X-100 solution for 1.5 hours and incubated with 3% blocking buffer for 1.5hr. The tissues were then incubated with secondary antibody, Alexa Fluor® 555 Goat Anti-Rabbit IgG (H+L) (Life Technologies), diluted 1:400 in 3% blocking buffer for 2hr on a rocking board. After incubation, the tissue fragments were washed with 1% Triton X-100 solution overnight at 4°C. On day 4, the tissues were co-stained with NucBlue Live Cell Stain ReadyProbes reagent (Life technologies, Hoechst 33342 Special Formulation) for nuclei stain and Lipidtox (HCS LipidTOX™ Deep Red neutral lipid stain) diluted 1:200 in PBS for lipid droplets stain for one hour. Then the tissue fragments were washed with PBS and mounted on glass slides in Prolong Gold Antifade mountant (Life Technologies) for imaging.

Whole body metabolic assessment and body composition analysis:

A standard 12 h light/dark cycle was maintained throughout the calorimetry studies for whole body metabolic assessment of mice. Twelve cages were contained in two separate temperature- and humidity-controlled temperature cabinets (Powers Scientific). Each mouse was individually housed in one of 24 center feeder cages in the Oxymax/CLAMS system and data (VO_2 and heat production or energy expenditure) was compiled using Oxymax/CLAMS software (Columbus Instruments). Feeders were filled completely with high fat diet (Research Diets). The mice were placed in the cages for one week at room temperature, after which the mice were removed from the chambers for 1 day while the cages were cleaned, food was re-loaded, and the internal temperature was adjusted to 8°C. Mice were placed back into their same cages for another week to collect measurements at 8°C. Mice were allowed to acclimate to the chambers for 2 days prior to the start of data collection. Data was collected using Oxymax/CLAMS software at 20 minute intervals for 7 consecutive days.

Body composition in terms of fat mass was measured in conscious animals by quantitative nuclear magnetic resonance spectroscopy (EchoMRI™; Echo Medical Systems). A system test is routinely performed at the beginning of each measurement day. The equipment is calibrated by scanning a calibration holder containing a known amount of fat to test the validity of measurement following user instructions. Mice were individually placed in an animal holder and into EchoMRI-100H. Whole body masses of fat, lean, free water, and total water were compiled by EchoMRI-100H and analyzed in GraphPad Prism7.

Supplementary Figure Legends:

Supplementary Figure 1. Image analysis for quantifying UCP1/lipid fraction and cell density quantitation for mouse tissues. **(A)** Original confocal slices (left) and UCP1 segmented images (right) of interscapular BAT and inguinal WAT tissue. The value indicates the algorithm output for UCP1 volume fraction of the particular stack. **(B)** Original confocal slices (left) and lipid segmented images (right) of interscapular BAT and inguinal WAT tissue. The value indicates the algorithm output for lipid volume fraction of the particular stack. **(C)** Original confocal slices (left) and nuclei segmented images (right) of interscapular BAT and inguinal WAT tissue. The value indicates the algorithm output for cell density of the particular stack.

Supplementary Figure 2. Optimization of browning media conditions from 1-3 weeks in both mouse (A-C) and human (D-F) tissues. Confocal z-stacks from whole-mount stained tissues were quantified for (A, D) UCP1 intensity, (B, E) UCP1 volume fraction, and (C, F) lipid volume fraction. Graphs display Mean +/- SEM; n=5-25 depending on sample. Statistical analysis conducted via one-way ANOVA followed by Tukey's multiple comparison's test. Stars indicate significance as compared to WAT tissue. ** indicate $p < 0.01$, *** indicate $p < 0.001$, **** indicate $p < 0.0001$.

Supplementary Figure 3. Human tissue analysis after 3 weeks of culture. Confocal z-stacks were quantified for **(A)** UCP1 intensity, **(B)** UCP1 volume fraction, **(C)** Lipid volume fraction for tissues cultured after 3 weeks. Plots display Mean +/- SEM; n=8 for each group. **** indicate $p < 0.0001$.

Supplementary Figure 4. Quantification of epididymal versus inguinal tissue browning. Tissue samples taken from two different mice and four representative tissues were used for each mouse (n=8). Error bars are SEM; ns indicates no significance (p = 0.5260 using Student's t-test).

Supplementary Figure 5. Body weight and metabolic assessment. **(A)** Body weight changes over duration of experiment. Arrows indicate stress-related weight loss due to moving animals into metabolic chambers, followed by additional weight loss after 1 week exposure to cold temperature (8° C). **(B)** VO_2 and **(C)** heat expenditure measurements taken 1 week post-implantation for animals receiving transplants of tissue browned in control media or browning media (n=8 for each group). Measurements taken at 20 minute intervals over the course of 6 days at room temperature followed by 6 days at 8ºC for VO_2 (left) and heat expenditure (right).

Supplementary Video Legends:

Supplementary Video 1: Confocal z-stack of whole mount mouse exBAT explant cultured in browning media for 3 weeks followed by 8 weeks re-implantation; fixed and stained with UCP1 antibody (red), Lipidtox (green), and Sytox nuclear stain (blue).

Supplementary Video 2: Confocal z-stack of whole mount mouse interscapular BAT tissue (positive control, uncultured); fixed and stained with UCP1 antibody (red), Lipidtox (green), and Sytox nuclear stain (blue).

Supplementary Video 3: Confocal z-stack of whole mount human WAT fragments cultured in browning media for 7 days; fixed and stained with UCP1 antibody (red), Lipidtox (green), and Sytox nuclear stain (blue).

Supplementary Video 4: Confocal z-stack of whole mount human WAT fragments cultured in control media for 7 days; fixed and stained with UCP1 antibody (red), Lipidtox (green), and Sytox nuclear stain (blue).

Supplementary Figures:

Supplementary Figure 1

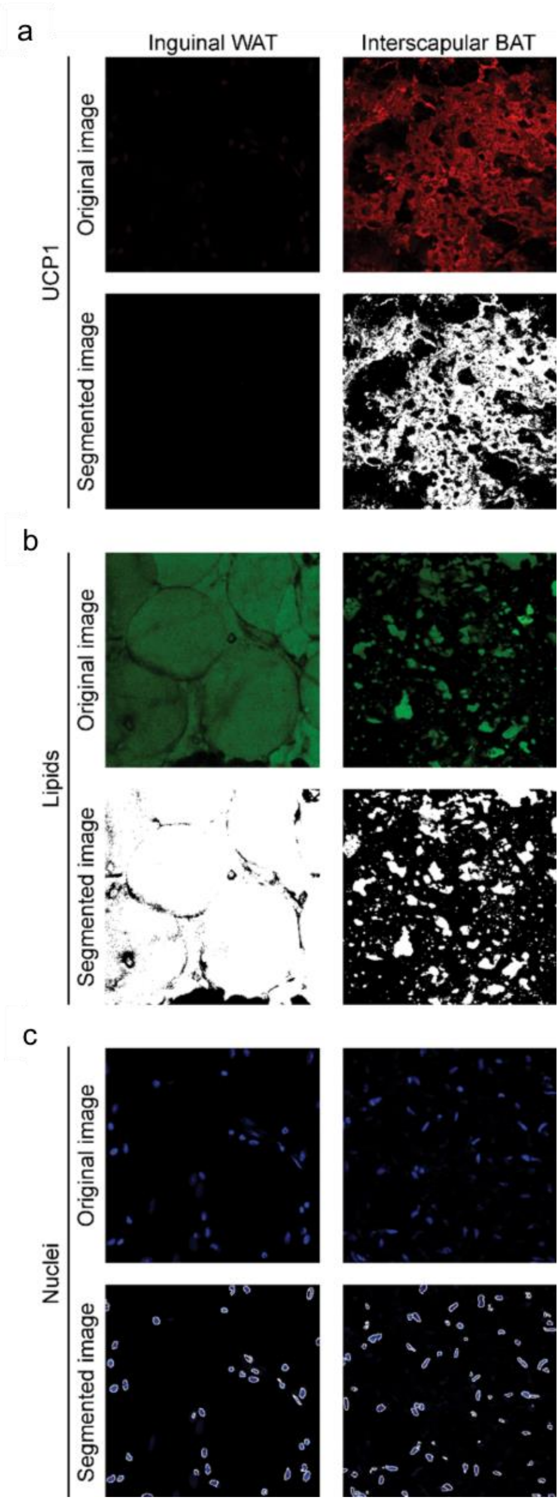

Supplementary Figure 2

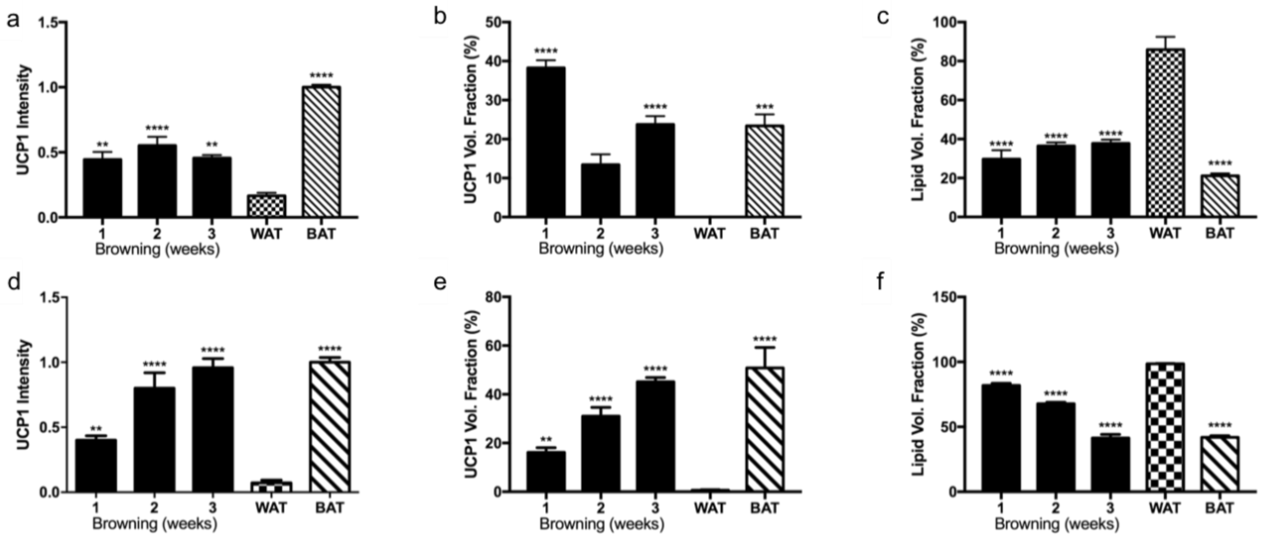

Supplementary Figure 3

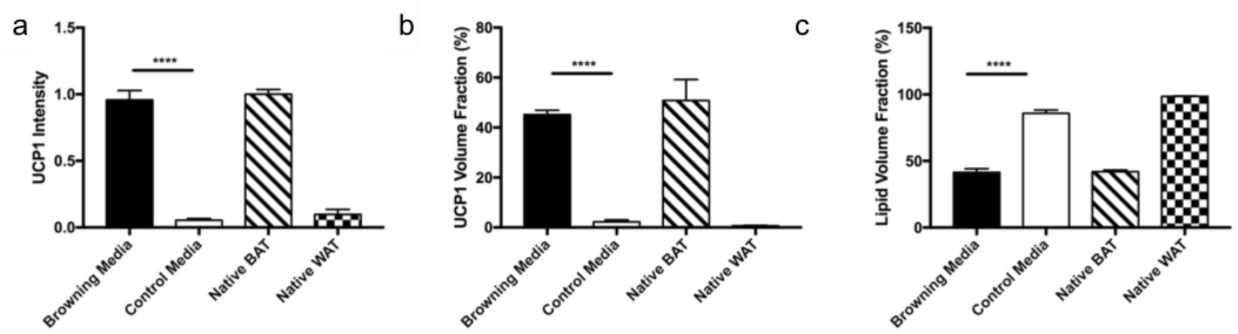

Supplementary Figure 4

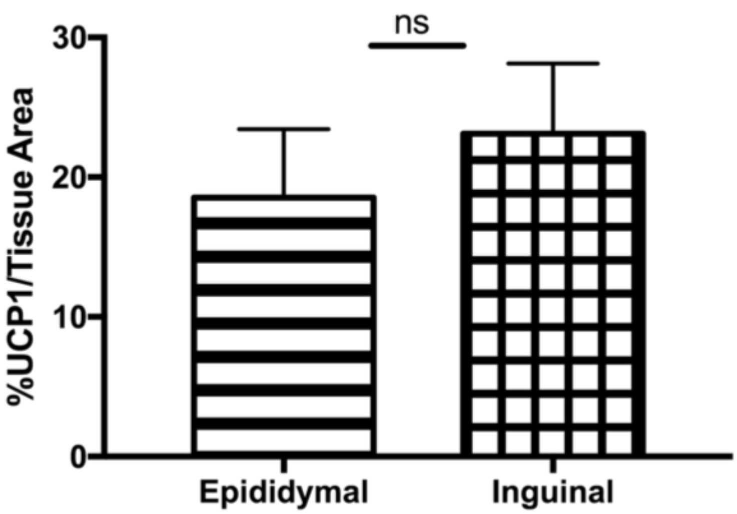

Supplementary Figure 5

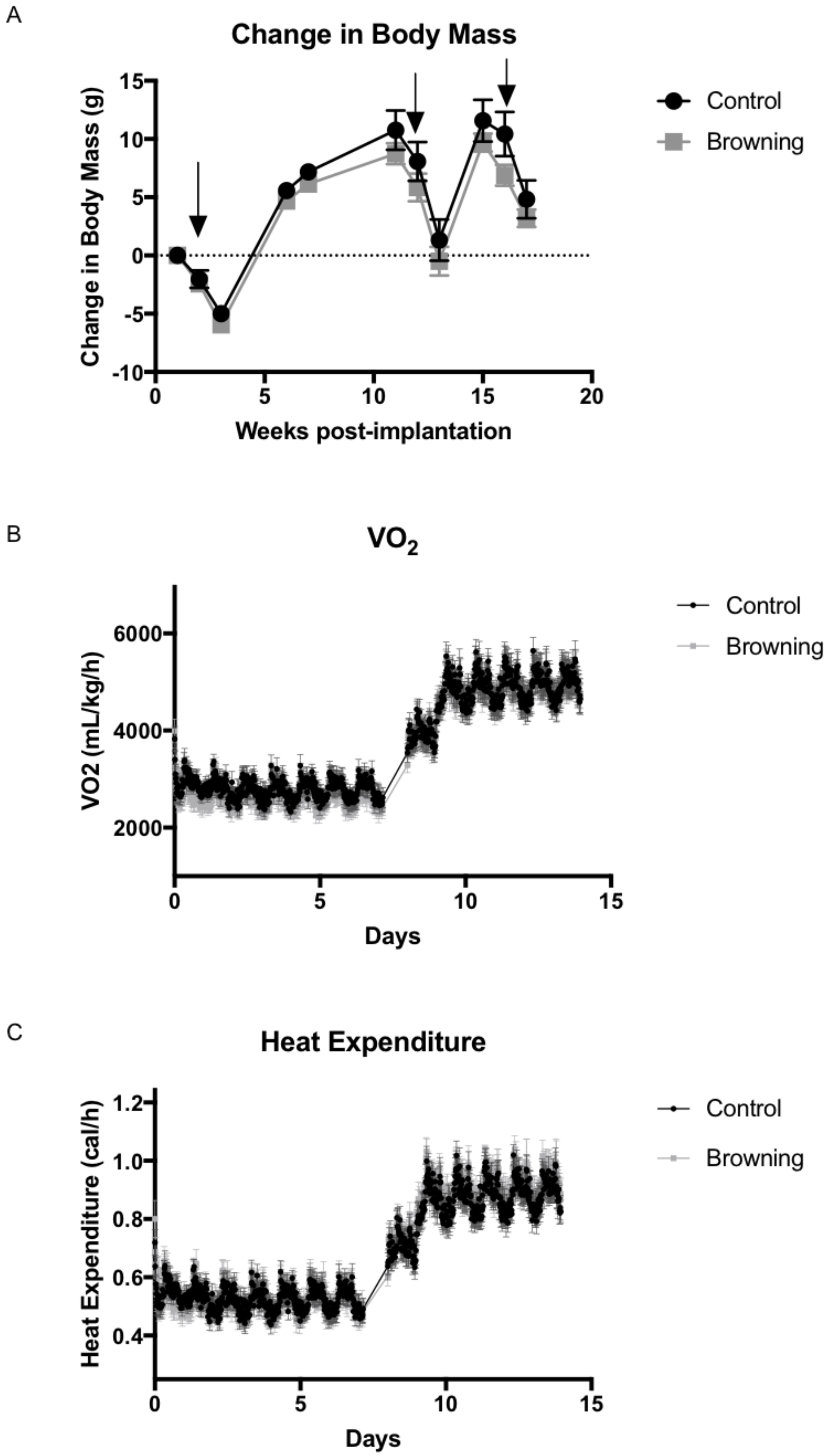

Supplement: Supplementary file 1 — Supplementary Information [file 41598_2018_25866_MOESM1_ESM.pdf]
